# A potential pathogenic association between periodontal disease and Crohn’s disease

**DOI:** 10.1172/jci.insight.148543

**Published:** 2021-12-08

**Authors:** Jin Imai, Hitoshi Ichikawa, Sho Kitamoto, Jonathan L. Golob, Motoki Kaneko, Junko Nagata, Miho Takahashi, Merritt G. Gillilland, Rika Tanaka, Hiroko Nagao-Kitamoto, Atsushi Hayashi, Kohei Sugihara, Shrinivas Bishu, Shingo Tsuda, Hiroyuki Ito, Seiichiro Kojima, Kazunari Karakida, Masashi Matsushima, Takayoshi Suzuki, Katsuto Hozumi, Norihito Watanabe, William V. Giannobile, Takayuki Shirai, Hidekazu Suzuki, Nobuhiko Kamada

**Affiliations:** 1 Division of Gastroenterology and Hepatology, Department of Internal Medicine, University of Michigan, Ann Arbor, Michigan, USA.; 2 Division of Gastroenterology and Hepatology, Department of Internal Medicine, Tokai University School of Medicine, Kanagawa, Japan.; 3 Division of Gastroenterology and Hepatology, Department of Internal Medicine, Tokai University School of Medicine Hachioji Hospital, Tokyo, Japan.; 4 Center for Preventive Medicine, Keio University School of Medicine, Tokyo, Japan.; 5 Division of Infectious Diseases, Department of Internal Medicine, University of Michigan, Ann Arbor, Michigan, USA.; 6 Department of Oral and Maxillofacial Surgery, Tokai University School of Medicine Hachioji Hospital, Tokyo, Japan.; 7 Department of Immunology, Tokai University School of Medicine, Kanagawa, Japan.; 8 Research Laboratory, Miyarisan Pharmaceutical Co. Ltd., Tokyo, Japan.; 9 Department of Oral Medicine, Infection, and Immunity, Harvard University School of Dental Medicine, Boston, Massachusetts, USA.

**Keywords:** Gastroenterology, Microbiology, Inflammatory bowel disease

## Abstract

Oral conditions are relatively common in patients with inflammatory bowel disease (IBD). However, the contribution of oral maladies to gut inflammation remains unexplored. Here, we investigated the effect of periodontitis on disease phenotypes of patients with IBD. In all, 60 patients with IBD (42 with ulcerative colitis [UC] and 18 with Crohn’s disease [CD]) and 45 healthy controls (HCs) without IBD were recruited for this clinical investigation. The effects of incipient periodontitis on the oral and gut microbiome as well as IBD characteristics were examined. In addition, patients were prospectively monitored for up to 12 months after enrollment. We found that, in both patients with UC and those with CD, the gut microbiome was significantly more similar to the oral microbiome than in HCs, suggesting that ectopic gut colonization by oral bacteria is increased in patients with IBD. Incipient periodontitis did not further enhance gut colonization by oral bacteria. The presence of incipient periodontitis did not significantly affect the clinical outcomes of patients with UC and CD. However, the short CD activity index increased in patients with CD with incipient periodontitis but declined or was unchanged during the study period in patients without periodontitis. Thus, early periodontitis may associate with worse clinically symptoms in some patients with CD.

## Graphical abstract

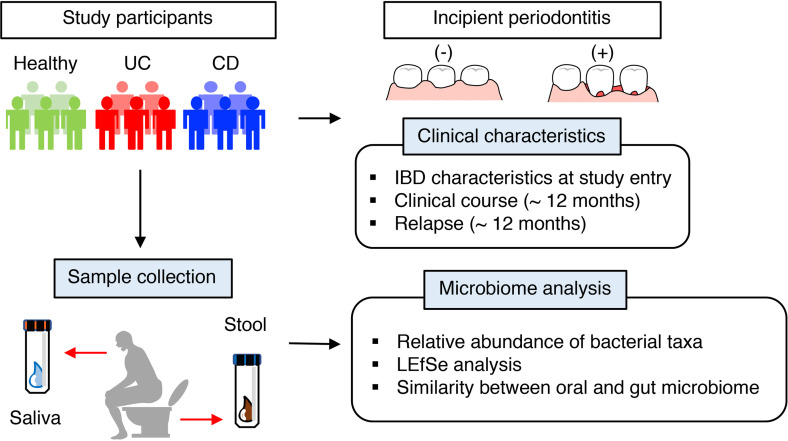

## Introduction

Inflammatory bowel diseases (IBD), including ulcerative colitis (UC) and Crohn’s disease (CD), are chronic inflammatory diseases, primarily involving the intestine. About one-third of patients with IBD develop extraintestinal manifestations (EIMs) in other organs, such as joints, skin, eyes, and the biliary tract (1, 2). The oral cavity is also a common site for EIMs in patients with IBD, especially CD (1). Salivary dysfunction and oral problems are often observed in patients with CD compared with healthy individuals (3). Likewise, the prevalence of periodontitis is significantly higher in patients with CD than in controls without IBD (4). Shared common pathological and inflammatory processes suggest an association between periodontitis and IBD. However, it is largely unclear if, and how, oral manifestations, such as periodontitis, influence intestinal inflammation in IBD.

The perturbed microbial community in the gut, so-called gut dysbiosis, is a hallmark of IBD (5). Genetic and environmental factors associated with the risk of IBD may drive the alteration of the gut microbial communities, enriching pathobionts and reducing beneficial bacteria (6, 7). The dysbiotic microbiota subsequently activates various immune pathways at the mucosal sites, thereby causing or exacerbating intestinal inflammation (8). Advances in next-generation sequencing technologies enable high-resolution and quantitative microbiome analysis in IBD. Using these techniques, several studies have identified bacterial taxa that are selectively enriched in patients with IBD (9, 10). Advanced microbiome analyses have reconfirmed the possible contribution of well-recognized IBD-associated pathobionts, such as members of the large family *Enterobacteriaceae*, including adherent-invasive *E*. *coli*. In addition, several previously underrecognized bacterial taxa have been identified as putative pathobionts. Notably, various studies have reported that oral bacteria, such as *Fusobacteriaceae*, *Pasteurellaceae*, and *Veillonellaceae*, are enriched in the mucosal tissues of patients with IBD (11, 12). The abnormal Th1/Th17-shifted immune activation observed in the mucosal tissues of these patients (13) suggests that ectopic colonization by some oral mucosa–derived bacteria may elicit the inflammatory immune responses in the gut mucosa. Indeed, *Klebsiella* species isolated from the saliva of patients with IBD harbor a potent Th1-inducing capacity when they colonize the colonic mucosa and provoke intestinal inflammation (14). Thus, gut colonization by certain oral bacteria, most likely oral disease–associated pathobionts, may contribute to IBD risk.

Despite recognizing the possible contribution of oral pathobionts to the pathogenesis of IBD, the precise mechanisms of how oral manifestations bridge the microbial oral–gut axis remain incompletely understood. In this regard, we have recently reported that periodontitis exacerbates colitis in mice (15). Oral inflammation induced by periodontitis results in expansion of oral *Klebsiella* and *Enterobacter* species, which are ingested and translocate to the gut (15). In the gut, these oral pathobionts may elicit inflammatory responses in the colonic mucosa (15). In parallel, oral pathobiont-reactive Th17 cells generated in the oral mucosa due to periodontitis migrate to the gut mucosa and aggravate colitis (15). Thus, oral inflammation can trigger ectopic gut colonization by oral pathobionts, thereby serving as a critical pathogenic factor that elevates the risk of IBD. In this context, patients with IBD display oral dysbiosis likely caused by oral conditions, including periodontitis (3, 11, 16). However, the effect of oral inflammation or dysbiosis on gut pathophysiology in human IBD remains elusive.

Here, we examined the oral health conditions and the oral microbiome of patients with IBD. The potential effect of periodontitis on conditions associated with intestinal involvement was also assessed. Furthermore, patients with quiescent disease at the time of study entry were prospectively monitored to elucidate the effect of periodontitis on disease relapse.

## Results

### Clinical characteristics.

**Figure 1:**
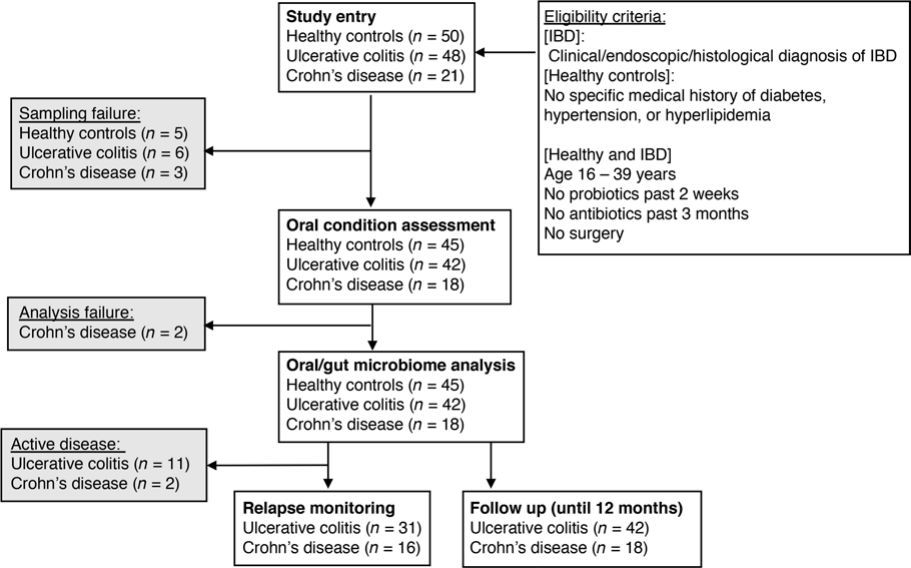
Participant inclusion and exclusion criteria and study flow diagram.

The patient inclusion criteria and study flow diagram are provided in Figure 1. A total of 69 patients with IBD (48 UC and 21 CD) and 50 healthy controls (HCs) without IBD were enrolled. Of all the participants, 5 HCs, 3 patients with CD, and 6 patients with UC were excluded due to their inability to provide saliva or stool samples. Baseline clinical characteristics and the oral and gut microbiome of the remaining 60 patients with IBD (42 UC and 18 CD) and 45 HCs were analyzed in this study. In addition, patients with IBD whose disease was quiescent at the time of entry (31 UC and 16 CD) were prospectively monitored for disease relapse for up to 12 months. Patient demographics are shown in Table 1 and Supplemental Table 1 (supplemental material available online with this article; https://doi.org/10.1172/jci.insight.148543DS1). We first focused on the age and smoking habits of the study participants, as these are significant factors associated with the periodontitis risk (17, 18). The average age of the patients with CD was slightly younger than that of the HCs (Table 1 and Supplemental Table 1). However, as all enrolled participants were between the ages of 16 and 39 years, the effects of periodontitis associated with aging (17) were considered to be negligible. Smoking habits were similar among the 3 study groups (Table 1 and Supplemental Table 1). We next investigated the oral condition of all study participants. There was no difference in the percentage of sites with bleeding on probing (BOP) among the 3 groups (Table 1 and Supplemental Table 1). The mean age of the patients in this study was young, which is understandable considering that IBD develops at a relatively early age. Further, it explains why none of the patients with IBD, nor the HCs, displayed severe periodontitis, which is rare in young individuals. Hence, we defined the presence of one or more periodontal pockets deeper than 4 mm as incipient periodontitis. Unlike previous studies (4), no difference was found in the prevalence of incipient periodontitis between HCs and patients with IBD (UC and CD) (Table 1 and Supplemental Table 1). The number of teeth was higher in patients with UC and CD compared with HCs (Supplemental Table 1). In patients with CD, the total number of teeth was higher in those individuals with incipient periodontitis [Perio (+)] compared with those without periodontitis [Perio (–)] (Supplemental Table 1). This difference was not observed in HCs or in patients with UC (Supplemental Table 1). The number of caries was not significantly different among the 3 groups. Only in patients with CD was the number of caries higher in Perio (+) patients compared with Perio (–) patients (Supplemental Table 1). No significant difference was found in oral humidity among HCs and patients with IBD (UC and CD) (Supplemental Table 1).

### Oral and gut microbiome analysis in IBD.

**Figure 2:**
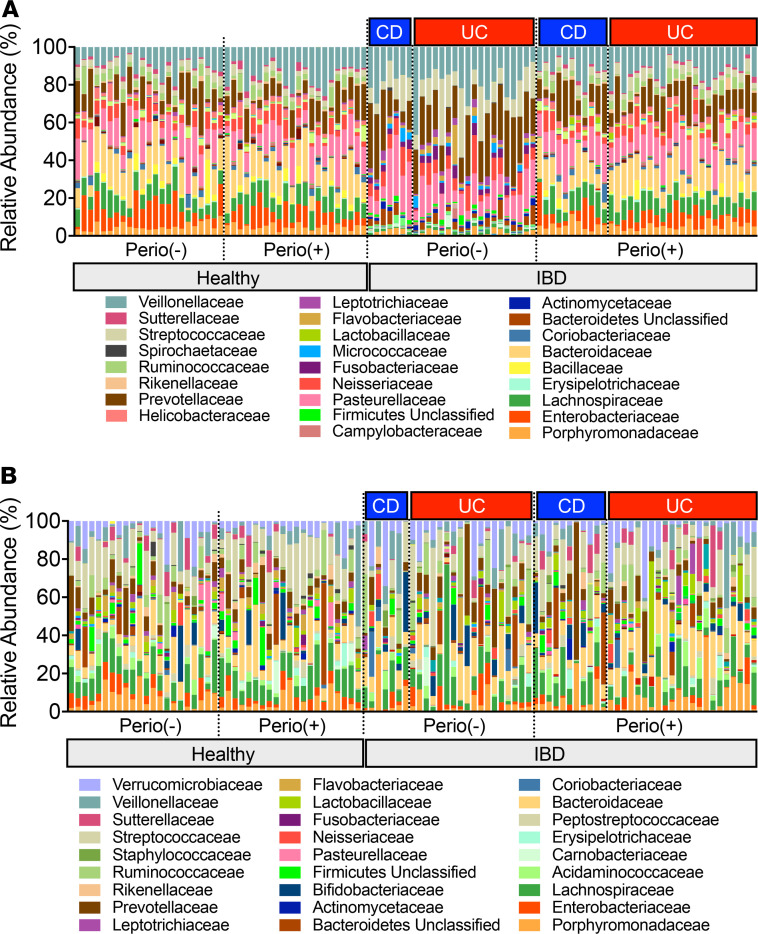
Oral and gut microbiome characterization. Microbial composition of the saliva and stool of healthy controls (HC) and patients with IBD (UC and CD) with and without incipient periodontitis was analyzed by 16S rRNA sequencing. The relative abundance of bacterial families is shown. (**A**) Oral microbiota (saliva). (**B**) Gut microbiota (stool).

We next analyzed the oral and gut microbiome in the patients with IBD and the HCs. Saliva and stool samples were isolated, and the microbial composition was assessed by 16S rRNA sequencing. Two patients with CD (CD-07 and CD-14) were excluded due to analysis failure caused by the quality of the 16S rRNA library of saliva microbiome samples (note that these patients were also excluded from the gut microbiome analysis). As previously reported (11), the oral microbial composition was altered in the patients with IBD (UC and CD) compared with HCs (Figure 2 and Supplemental Figure 1). Some bacterial taxa that are known to be associated with oral diseases, such as *Porphyromonadaceae* OTU0100, *Porphyromonas* OTU0018, and *Leptotrichia* OTU0037 (19, 20), were enriched in patients with UC compared with HCs (Supplemental Figure 1). However, different species and strains of bacteria in the same family (i.e., different operational taxonomic unit [OTU]) were also overrepresented in HCs (*Porphyromonadaceae* OTU0031 and OTU0162) (Supplemental Figure 1). Therefore, the accumulation of oral pathobionts may not be a specific change observed in patients with UC. In patients with CD, *Fusobacterium* OTU0043 and *Streptococcus* OTU0002, which likely contribute to oral disease, were significantly enriched compared with HCs (Supplemental Figure 1). On the other hand, levels of other potential oral pathobionts, such as *Enterobacteriaceae* and *Campylobacter*, were somewhat higher in HCs than in patients with CD (Supplemental Figure 1). In the stool samples, consistent with findings in previous reports (12, 21), we observed that some taxa associated with putative oral pathobionts accumulated in the gut of patients with IBD (e.g., *Prevotella*, *Porphyromonadaceae*, *Neisseria*, and *Veillonella* in UC and *Prevotella*, *Porphyromonadaceae*, and *Atopobium* in CD) compared with HCs (Supplemental Figure 1).

To address the impact of incipient periodontitis on the composition of the oral microbiome, we identified bacterial taxa enriched in the presence of periodontitis. Consistent with previous studies (22), periodontitis altered the oral microbiome in HCs, as shown by the accumulation of taxa associated with oral disease (e.g., *Prevotella*). When comparing Perio (–) and Perio (+) patients with IBD, bacterial taxa not enriched in Perio (+) HCs were found to be accumulated (e.g., *Pasteurellaceae*, *Neisseria*, and *Veillonella* in UC and *Porphyromonadaceae*, *Alistipes*, and *Enterobacteriaceae* in CD) (Supplemental Figure 2). Thus, HCs and patients with IBD may have slightly distinct oral microbiome at baseline and after the development of periodontitis. However, no specific pathogenic changes were observed in patients with IBD. The presence of deep pockets displayed a marginal effect on the gut microbiome compared with the oral microbiome. In HCs, no difference was observed between Perio (–) and Perio (+) individuals (Supplemental Figure 2). In patients with CD, *Alistipes* were enriched in Perio (+) compared with Perio (–) individuals (Supplemental Figure 2). No significant enrichment of bacteria was measured in Perio (+) patients with UC (Supplemental Figure 2).

As the family and genus level taxonomic resolution limits the prediction of the pathogenic potential of bacteria, we examined the status of IgA-coating of oral bacteria. Given that potential pathobionts harbor potent immunogenicity and are therefore highly coated by IgA, bacterial IgA-coating can be used to identify pathobionts (23). In this regard, we measured the abundance of oral bacteria that were highly coated by IgA in the saliva of HCs and patients with IBD. As shown in Supplemental Figure 3, significant populations of oral bacteria were coated by IgA. However, there were no differences between HCs and patients with IBD in the abundance of IgA-coated bacteria in saliva. IgA-coated bacteria were significantly enriched in the gut microbiota of patients with CD compared with HCs (Supplemental Figure 3). In this data set, we did not observe the enrichment of IgA-coated bacteria in the gut of patients with UC (Supplemental Figure 3).

### Increased ectopic gut colonization by oral bacteria in IBD.

**Figure 3:**
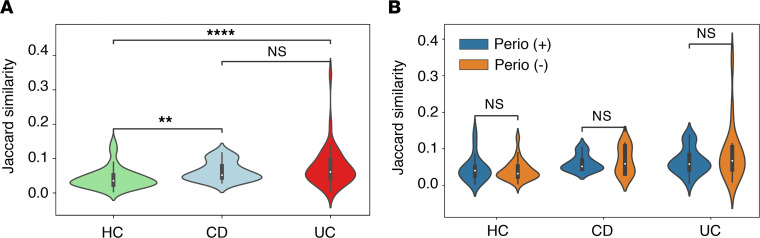
Similarity between the oral and gut microbiome. The similarity in bacterial taxa between the saliva and stool of individual patients was analyzed using Jaccard similarity. (**A**) The oral and gut bacterial similarity in HCs and patients with UC and CD. (**B**) The oral and gut bacterial similarity between HCs and patients with IBD with or without incipient periodontitis. *P* values by Student’s *t* test are shown. **P < 0.01; ****P < 0.0001.

To validate the extent to which oral bacteria ectopically colonize the gut of patients with IBD, we assessed the species of bacteria shared between the oral and gut microbiome in paired patients. Using Jaccard similarity, equivalent to the fraction of 16S rRNA V4 region sequence variants shared between the gut and oral microbiome, we found that in both patients with UC and those with CD, the gut microbiome was significantly more similar to the oral microbiome than in the HCs (Figure 3A). This was true whether or not the individual had periodontitis and regardless of disease state (Figure 3B). Thus, the increase in ectopic gut colonization by oral bacteria in patients with IBD may be due to gut dysbiosis. Unlike a known animal model of periodontitis-colitis (15), periodontitis did not further enhance the gut colonization by oral bacteria.

### Early periodontitis and clinical outcomes.

**Figure 4:**
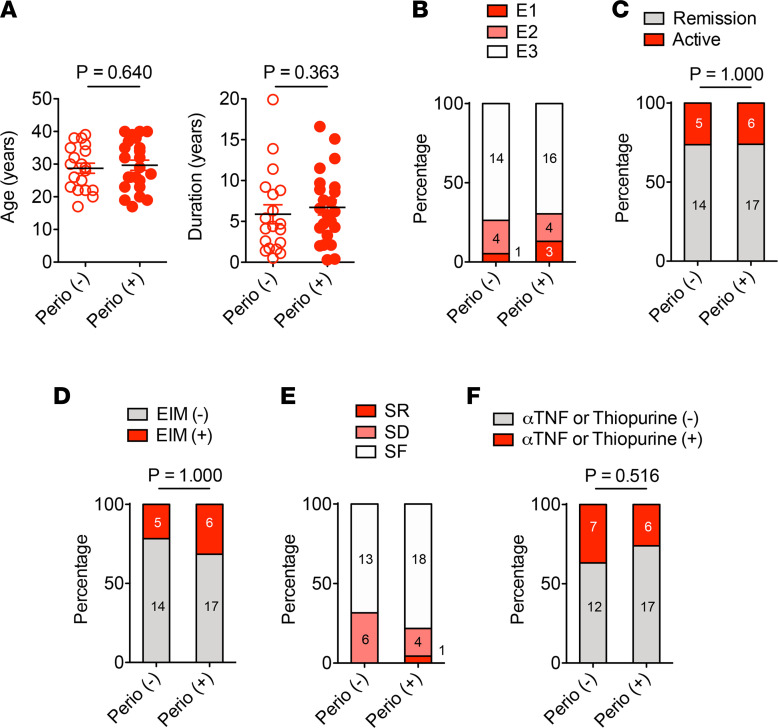
UC characteristics in patients with or without incipient periodontitis. (**A**) Age and disease duration at study entry. (**B**) Disease extent at diagnosis. E1, proctitis; E2, left sided; E3, extensive. (**C**) Disease activity. (**D**) Extraintestinal manifestations (EIM). (**E**) Response to steroid therapy. SR, steroid refractory; SD, steroid dependent; SF, steroid free. (**F**) The number of patients who received thiopurine or anti-TNF therapy. (**A**) Results are shown as mean ± SD. Dots indicate individual participants, as do numbers within bars. *P* values by Mann-Whitney *U* test (**A**) and Fisher exact test (**B–F**) are shown.

To examine the possible influence of early periodontitis on the intestinal involvement in patients with IBD, we assessed the clinical conditions of the patients with IBD with and without periodontitis. In patients with UC, no significant difference was found in age and disease duration between Perio (–) and Perio (+) individuals (Figure 4A and Supplemental Table 2). Most patients with UC were diagnosed with extensive (E3) colitis, and several had left-sided (E2) colitis or proctitis (E1) (Figure 4B and Supplemental Table 2). The percentage of patients with remission (defined as a partial Mayo score ≤2) was similar in the 2 groups (Figure 4C and Supplemental Table 2). Five of 19 Perio (–) and 6 of 23 Perio (+) patients with UC had EIMs (Figure 4D and Supplemental Table 2). The fraction of steroid-dependent and -refractory patients was similar between the Perio (–) and Perio (+) groups (Figure 4E and Supplemental Table 2). Anti-TNF agents and thiopurines — either monotherapy or combination therapy — are often used for induction therapy and maintenance therapy for moderate-to-severe disease (24–26). The percentage of patients who received this type of therapy was similar between Perio (–) and Perio (+) patients with UC (Figure 4F and Supplemental Table 2). Thus, the presence of periodontitis may not affect the severity of gut inflammation in patients with UC.

**Figure 5:**
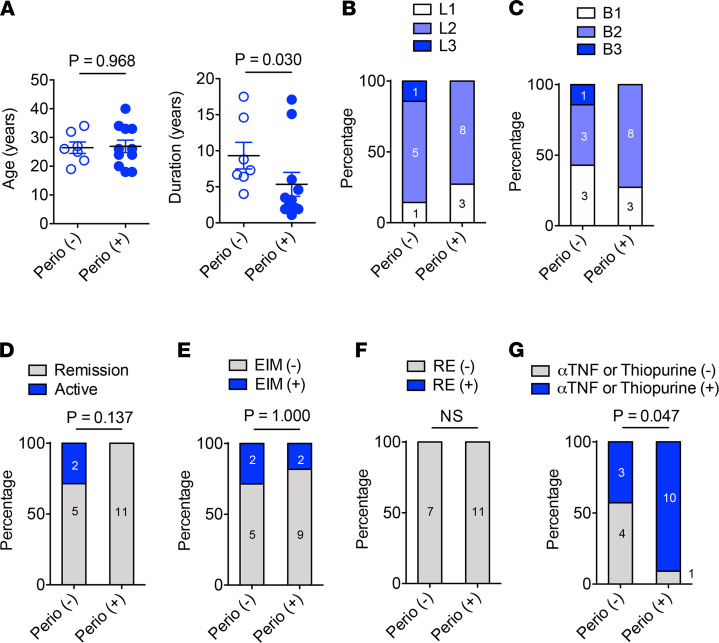
CD characteristics in patients with or without incipient periodontitis. (**A**) Age and disease duration at study entry. (**B**) Disease location at diagnosis. L1, terminal ileum; L2; colon; L3, ileocolonic. (**C**) Disease behavior. B1, nonstricturing, nonpenetrating; B2, stricturing; B3, penetrating. (**D**) Disease activity. (**E**) Extraintestinal manifestations (EIM). (**F**) Resistance (RE) to biologic therapy. (**G**) The number of patients who received thiopurine or anti-TNF therapy. (**A**) Results are shown as mean ± SD. Dots indicate individual participants, as do numbers within bars. *P* values by Mann-Whitney *U* test (**A**) and Fisher exact test (**B–G**) are shown.

In patients with CD, disease duration was significantly shorter in Perio (+) patients than Perio (–) patients, although the mean age of patients with CD was not different between Perio (–) and Perio (+) individuals (Figure 5A and Supplemental Tables 1 and 2). Most patients had isolated colonic (L2) disease (Figure 5B and Supplemental Table 2). The fraction of patients with complicated CD (B2 [structuring] or B3 [penetrating] disease) was similar between groups (Figure 5C and Supplemental Table 2). Most of the patients with CD enrolled in this study were in remission (defined as a as short CD Activity Index [sCDAI ≤4), except for 2 patients who did not have periodontitis (Figure 5D and Supplemental Table 2). Two of 7 Perio (–) and 2 of 11 Perio (+) patients with CD had EIMs (Figure 5E and Supplemental Table 2). While response to biologics was good, the fraction of patients who required biologics or thiopurines to control disease was higher in the Perio (+) group compared with the Perio (–) group (Figure 5, F and G, and Supplemental Table 2). In Japan, biologics are typically reserved for disease refractory to thiopurines. Thus, the fact that Perio (+) patients had shorter duration of disease, but relatively higher use of biologics may imply that the Perio (+) patients might have more difficult-to-control disease.

### Early periodontitis may have some effect on disease course of CD.

**Figure 6:**
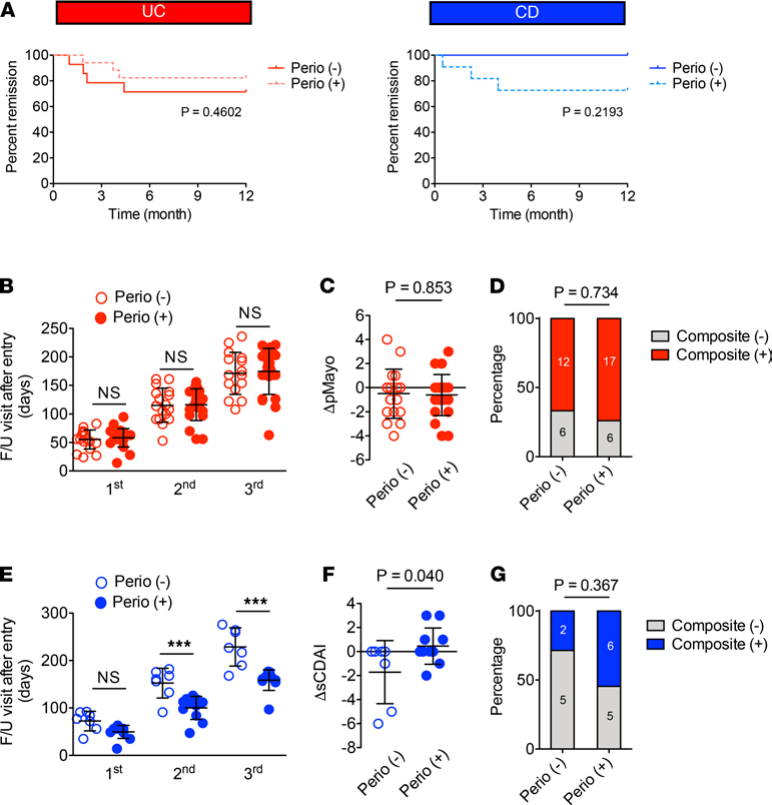
Effect of incipient periodontitis on IBD. (**A**) Patients with IBD who were in remission (i.e., pMayo score ≤2 for UC cases and sCDAI ≤4 for CD cases) at the time of study entry were prospectively monitored for disease relapse for up to 12 months (31 UC and 16 CD). The percentage of patients who maintained remission is shown. (**B**) Interval between follow-up visits (i.e., days from initial visit) in UC. (**C**) Change in pMayo score from the initial visit to the last visit (ΔpMayo) in UC. (**D**) The percentage of the composite (+) patients with UC. (**E**) Interval between follow-up visits (i.e., days from initial visit) in CD. (**F**) change in sCDAI score from the initial visit to the last visit (ΔsCDAI) in CD. (**G**) The percentage of the composite (+) patients with CD. (**B**, **C**, **E**, and **F**) Results are shown as mean ± SD. Dots indicate individual participants. *P* values by the log-rank test (**A**), 1-way ANOVA with the Bonferroni test (**B** and **E**), Student’s *t* test (**C** and **F**), and Fisher exact test (**D** and **G**) are shown.

In order to determine whether the presence of periodontitis is prognostic in CD, we calculated the risk of relapse in the Perio (+) and Perio (–) groups. Of 31 inactive patients with UC, 14 were Perio (–) and 17 were Perio (+) (Supplemental Table 2). Of 16 inactive patients with CD, 5 were Perio (+) and 11 were Perio (+) (Supplemental Table 2). In the UC Perio (–) group, 4 patients experienced disease relapse within 12 months (30, 57, 64, and 134 days after study entry) (Figure 6A and Supplemental Table 2). In the Perio (+) group, 3 patients with UC had a relapse within 12 months (56, 113, and 125 days after study entry) (Figure 6A and Supplemental Table 2). In the Perio (+) patients with CD, about 30% (3 of 11) experienced disease relapse (15, 69, and 120 days after study entry), while all Perio (–) patients with CD (5 of 5) maintained remission for 12 months (Figure 6A and Supplemental Table 2). Although collectively these results were not statistically significant, they suggest a possibility that incipient periodontitis may have some effect on the risk of relapse in patients with CD.

To further investigate the impact of incipient periodontitis on disease outcome in IBD, we retrospectively analyzed the clinical course of patients (Supplemental Table 3). In patients with UC, the time between follow-up visits did not differ in the Perio (+) and Perio (–) groups (Figure 6B). In addition, the change in partial Mayo score between the initial visit (study entry) and the last visit (ΔpMayo) was not different between the Perio (+) and Perio (–) groups (Figure 6C). In addition to these analyses, we developed a composite outcome of any increase in the partial Mayo score or a flare to determine if periodontitis correlates with the clinical course of UC. No significant difference was measured between the presence or absence of periodontitis (Figure 6D).

In patients with CD, the follow-up interval was significantly shorter in Perio (+) patients than Perio (–) patients (Figure 6E). The sCDAI increased in Perio (+) patients with CD but declined in Perio (–) patients with CD over the study period (Figure 6F). Regarding the composite outcome, 2 patients in the Perio (–) group reached the composite endpoint of an increase in sCDAI or a flare, while 6 patients in the Perio (+) group experienced either a flare (*n* = 3) or an increase in sCDAI (*n* = 3) (Figure 6G). Thus, there was a trend toward more active disease in the Perio (+) group over the study period (*P* = 0.37), despite a trend toward a shorter follow-up interval relative to the Perio (–) group.

To identify specific oral bacterial species accumulated in the gut of relapsed patients, we analyzed the similarity between the oral and gut microbiome of patients with IBD with or without relapse. As few patients experienced disease relapse during the 12-month follow-up period, we combined the UC and CD patient data for this analysis. Interestingly, although the degree of overlap between the gut and oral microbiome was similar between those with or without periodontitis and with or without relapse, the specific microbial species shared between the gut and oral microbiome seemed to vary by relapse. Again, though this finding is limited by small sample size, *H*. *parainfluenzae*, *S*. *parasanguinis*, and *S*. *odontolytica* were shared between the gut and oral microbiome in nearly all observed participants with IBD who experienced relapse. Likewise, nearly all individuals who relapsed and who had periodontitis shared *V*. *parvula* and *Gemella* spp. between their gut and oral microbiome (Supplemental Figure 4).

## Discussion

In this study, we demonstrate an increase in gut colonization by oral bacteria in both patients with UC and those with CD compared with HCs. Although incipient periodontitis does not accelerate ectopic gut colonization by oral bacteria, incipient periodontitis may have some effect on gut disease in CD. In patients with CD, the sCDAI increased in Perio (+) patients and decreased in Perio (–) patients over the study period (ΔsCDAI). In addition, the follow-up interval was significantly shorter in Perio (+) patients than Perio (–) patients. Given that all patients received care at a single center, it is plausible that all patients had similar care pathways in regards to access to biologics, compared with larger institutions, multicenter institutions, or public versus private centers, which would be expected to have larger variance in biologic prescribing patterns. Further, new anti-TNFs were not approved in Japan over the study period of 2018–2019. Thus, a plethora of new or more effective drugs was not available between the study start and end. Consideration of these points suggests that patients probably received consistent treatment. Therefore, the shorter follow-up intervals and increased ΔsCDAI may imply more difficult-to-control disease. However, further studies along with a larger sample size will be required to draw a more firm conclusion that periodontitis affects clinical outcomes of CD.

Although previous studies have reported that patients with IBD display a higher prevalence of oral disease, including periodontitis (27, 28), we did not observe this tendency in our cohort. This may be because our study population was relatively younger. Most oral conditions, such as BOP percentage, the number of caries, and the degree of oral moisture, were not significantly different between the HCs and the patients with IBD (UC and CD). Interestingly, the number of teeth was significantly higher in the patients with UC and CD compared with the HCs (*P* < 0.05 [HC vs. UC or CD] by 1-way ANOVA followed by Bonferroni’s post hoc test). This was due to the number of remaining wisdom teeth. Wisdom teeth, which are the third and final set of molars (29), are often misaligned, causing periodontal problems with adjacent teeth (30, 31). The extraction of wisdom teeth is considered a clinical option that maintains oral health (29). Although it remains entirely unknown why fewer patients with IBD, at least in this cohort, had experienced wisdom teeth removal, the periodontal problems caused by retainment of the third set of molars may affect the intestinal involvement in IBD. It is worth noting that wisdom teeth usually emerge between 17 and 25 years of age, which overlaps in time with the main peak age of the onset of IBD. Thus, although further study is required to prove the precise connection, it is possible that the presence of wisdom teeth contributes to the pathogenesis of IBD.

It has been reported that more patients with IBD display oral dysbiosis compared with HCs (11). Consistently, we observed differences in the composition of the salivary microbiota between the HCs and the patients with IBD. The presence of periodontitis enriched the oral pathobionts in HCs. Periodontitis had a more dramatic effect on the oral microbiome in patients with IBD than HCs. Of note, the *Enterobacteriaceae* family was significantly enriched in patients with CD with periodontitis compared with those without. Given that the amassed *Enterobacteriaceae* in the oral cavity is associated with the increased susceptibility to colitis in mice (15), these bacteria may also contribute to the risk of CD in humans. However, further studies are required to examine the extent to which the enriched bacterial species and strains harbor colitogenic capacity in humans. Likewise, it is noteworthy that we did not observe the expansion of classic periodontal pathobionts in the individuals with periodontitis or in the HCs. This may be related to the patient selection, as the participants in this study were relatively younger and therefore had developed only mild (i.e., incipient) periodontitis. It is also possible that saliva samples may have a different microbial composition than periodontal pockets.

We found that in both patients with UC and those with CD, the similarity between the oral and gut microbiota was significantly higher than that in the HCs. This result suggests that ectopic gut colonization by oral bacteria is increased in patients with IBD. As most of the patients with IBD were in remission when the oral and gut microbiome samples were collected, it is plausible that patients with IBD are predisposed to the colonization by oral bacteria, regardless of the presence of active disease that may disrupt gut-resident microbiota. Unlike the animal periodontitis-colitis model (15), the presence of incipient periodontitis did not further enhance the gut colonization by oral bacteria in patients with IBD. However, incipient periodontitis may have a negative effect on the clinical course of IBD, especially in CD. This implies that periodontitis may affect host immune activation, which, in turn, contributes to gut disease. In this regard, we have reported the immunological connection between the oral and gut mucosa in animal models of IBD. In the mouse periodontitis-colitis model, periodontitis results in the emergence of pathogenic Th17 cells in the oral cavity (15). These de novo–generated pathogenic Th17 cells transmigrate to the gut mucosa, where they react to the ectopically colonized oral pathobionts and contribute to intestinal inflammation. Thus, it is possible that immune cells, such as Th17 cells, arise during periodontitis and contribute to gut inflammation in cooperation with oral pathobionts in patients with IBD. However, direct assessment of oral T cell migration to the gut is difficult in humans. Alternatively, analysis of the reactivity of circulating Th17 cells to oral pathobionts may, in part, address this question. If periodontitis induces the differentiation of gut-tropic pathogenic T cells in humans, as occurs in mice, anti-integrin therapy may be more effective in patients with IBD who have periodontitis. Thus, the presence of periodontitis may identify a subpopulation of patients who respond to certain IBD therapies, such as anti-integrin therapy.

This study, however, has limitations. One limitation is the small size of samples, particularly of the subgroups, such as the numbers of patients who experienced relapse. In fact, the majority of IBD outcomes evaluated in this study were not statistically significant due to the sample size. In addition, the criteria used to diagnosis periodontal disease in such a young population of individuals required the consideration of IBD conditions; the young age of the population indicated that patients had a very early form of periodontitis. As such, the population does not represent a typical cohort; individuals with more severe forms of disease are represented in later decades of life. This may be a reason why we did not observe the expansion of classic periodontal pathobionts in the individuals with periodontitis. Thus, the populations used in this study possessed inherent limitations for the evaluation of the influence of periodontitis on IBD or the impact of ectopic gut colonization by classic periodontal pathobionts. Another limitation is the heterogeneity of the patients enrolled (i.e., a mix of treatment durations, diagnoses, disease activity, and treatments). Thus, further studies will be required to determine if the observations in this study are reproducible and statistically significant using a more homogeneous patient cohort (e.g., treatment-naive patients or patients with active disease who are beginning a new treatment), along with a larger sample size. Although, some clinical parameters, such as the ΔsCDAI, revealed a potential effect of early periodontitis on intestinal inflammation in patients with CD, the presence of incipient periodontitis did not significantly affect the clinical outcomes of patients with UC and CD in this study, given the aforementioned limitations (small sample size, patient heterogeneity, and mild extent of periodontitis). Despite its limitations, this study shows more comprehensive microbial profiles of patients with UC and CD than other reported studies to our knowledge and, hence, provides valuable insight, as a proof of concept, into the possible role of periodontal inflammation and oral dysbiosis in the clinical course of IBD.

## Methods

### Patients.

Sixty-nine patients with IBD (48 UC and 21 CD) and 50 HCs without IBD were enrolled in the study at Tokai University Hachioji Hospital in Tokyo, Japan. Eligible participants aged 16–39 years were enrolled to exclude age-related periodontal disease. HCs had no specific medical history, including diabetes, hypertension, or hyperlipidemia. The clinical diagnosis of UC or CD was based on established clinical, endoscopic, and histological criteria. Patient demographics, disease characteristics, clinical symptoms, EIMs of IBD, associated primary sclerosing cholangitis, and medication use were extracted from the medical records. None of the participants took any probiotics within the last 2 weeks or any antibiotics within the last 3 months prior to study entry. Patients who had undergone surgical resection for intestinal lesions were excluded. Disease relapse was assessed by the partial Mayo score for UC or the sCDAI for CD. Clinical remission was defined as a partial Mayo score of ≤2 points for UC cases and a sCDAI of ≤4 points for CD cases. Patients were consecutively enrolled in 2018 at a single center and followed through 2019 by their treating health care provider.

### Patient treatment, follow-up, and outcomes.

Clinical management, including medication use, was based on the clinical judgment of the treating provider. In general, either thiopurines or biologics are used as first-line therapy for IBD. However, providers often use 5-aminosalicylic acid as first-line therapy for UC and thiopurines as first-line therapy for CD before prescribing biologics. A disease flare was defined as increasing symptoms: a pMayo score >2 for UC or sCDAI >4 for CD, and requiring a change of medication, whether an increase in current medication or the use of corticosteroids, as judged by the treating physician. Corticosteroids are often used to treat a flare. Patients are considered steroid dependent if they experience a flare when tapering a medication, and steroid refractory if their flare does not respond to high-dose steroids (1–1.5 mg/kg for 1–2 weeks). Given that this is an exploratory, pilot study that could not be completely powered, we constructed a composite endpoint of two components: (a) any increase in the pMayo score throughout the study period or a flare for UC and (b) any increase in the sCDAI or a flare for CD. Patients with quiescent disease at the time of study entry (31 UC and 16 CD) were prospectively monitored for disease relapse up to 12 months after enrollment.

### Oral examination and biospecimen collection.

We collected saliva and stool samples from 60 patients with IBD (42 UC and 18 CD) and 45 HCs. Saliva and stool samples were collected at the same time. All 60 patients with IBD and 45 HCs underwent a consultation with a dental specialist at the Department of Oral and Maxillofacial Surgery, Tokai University Hospital. Oral conditions, including BOP, caries, and oral humidity in the oral cavity, were evaluated. Oral moisture was measured at the lingual mucosa surface using an oral moisture–checking device (Mucus, Life Co.) (32). Oral moisture levels ranged from 0 to 99.9 degrees: normal, ≥29.6 degrees; borderline dry mouth, 28.0–29.5 degrees; and dry mouth, ≤27.9 degrees. We defined the presence of 1 or more periodontal pockets deeper than 4 mm as incipient periodontitis.

### Microbiome analysis.

For oral and gut microbiome analysis, saliva and stool samples were obtained from all participants (i.e., the patients with IBD and the HCs without IBD. Bacterial DNA was extracted using a modified protocol from the DNeasy Blood & Tissue Kit (Qiagen) (33). Microbiome analysis was performed at the Microbial Systems Molecular Biology Laboratories at the University of Michigan. 16S rRNA gene libraries were constructed using primers specific to the V4 region and processed by Illumina MiSeq. Sequences were curated using the community-supported software program mothur (34). Sequences were assigned to OTUs using a 0.03 cutoff and classified against the Ribosomal Database Project 16S rRNA gene training set (version 9; http://rdp.cme.msu.edu/index.jsp), using a naive Bayesian approach with an 80% confidence threshold. Curated OTU sequence data were converted to relative abundance ± SEM. Linear discriminant analysis effect size (35) was used to identify bacterial taxa that were differentially abundant with >0.15% abundance, biological consistency, and the greatest effect size. In some experiments, sequence variants were generated using DADA2 (36) and then phylogenetically placed on a custom reference set (37, 38).

### Flow cytometric analysis of salivary and fecal bacteria.

Eighteen participants from each group (HC, UC, and CD) who had a sufficient amount of both the saliva and stool samples were selected, and their samples were used for IgA-coated bacteria analyses. As previously described (23, 39), stool samples were suspended in ice-cold PBS (100 mg/mL) and then centrifuged (100*g*, 15 minutes, 4°C) to remove large debris. Saliva samples (0.1 mL) were processed similarly. The supernatants, which contain bacteria, were harvested and centrifuged for 5 minutes (8000*g*, 4°C). Bacterial pellets were then resuspended in ice-cold staining buffer (PBS containing 0.5% BSA). Bacterial suspensions were incubated in a staining buffer containing 20% Normal Mouse Serum (Rockland) for 20 minutes. Antibody-coated bacteria were then stained with PE-labeled anti-human IgA (1:50, Miltenyi Biotec, clone IS11-8E10; mouse IgG1κ) or PE-labeled isotype control (IgG1κ; BioLegend) for 30 minutes. After staining, samples were washed with staining buffer and fixed with 4% paraformaldehyde for 15 hours at 4°C. After washing twice with the staining buffer, samples were suspended in PBS containing DAPI (12.5 μg/mL, MilliporeSigma). Samples were analyzed by flow cytometry (FACS Fortessa, BD Biosciences).

### Data availability.

The microbiome data in this study are available at the NCBI Sequence Read Archive, under BioProject PRJNA684508 (saliva) and PRJNA684584 (stool).

### Statistics.

Statistical analyses were performed using Prism 5.0 (GraphPad Software). Differences between 2 groups were compared using the 2-tailed Student’s *t* test (parametric) or the Mann-Whitney *U* test (nonparametric). To compare more than 3 groups, statistical analysis was performed using 1-way ANOVA (parametric) or the Kruskal-Wallis test (nonparametric), followed by Bonferroni’s correction for parametric samples or Dunn’s test for nonparametric samples as a post hoc test. To determine a significant relationship between 2 categorical variables, Fisher’s exact test was used. All levels of significance were set at *P* < 0.05.

### Study approval.

The study design was reviewed and approved by the Medical Ethics Committee at Tokai University (no. 17R-364). Written informed consent was received from all participants prior to inclusion in the study. All participants were followed in our outpatient clinic within the research period from April 2018 to March 2020.

## Author contributions

JI, H. Ichikawa, and NK conceived and designed the experiments. MT collected all the dental data. WVG contributed to the analysis of the dental data. S. Kitamoto, MGG, and JLG performed oral and gut microbiome analysis, with help from HNK, AH, KS, and SB. JI, RT, and KH analyzed IgA-coating bacteria. H. Ichikawa contributed to the collection of the clinical data, with help from MK, JN, ST, H. Ito, S. Kojima, KK, MM, NW, T. Suzuki, HS, and T. Shirai. JI, H. Ichikawa, S. Kitamoto, and NK analyzed the data. JI and NK wrote the manuscript with contributions from all authors.

## Supplementary Material

Supplemental data

## Figures and Tables

**Table 1:**
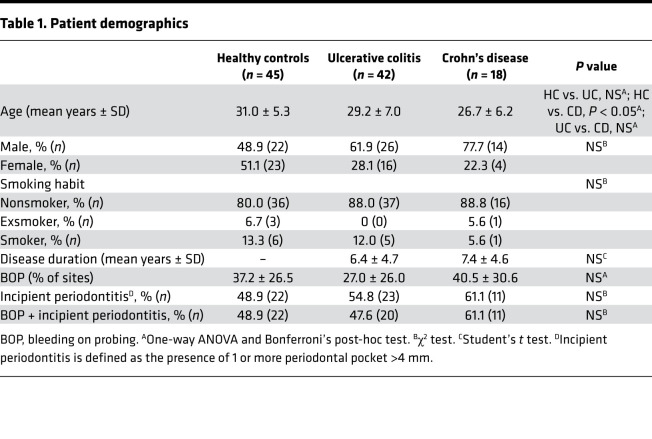
Patient demographics

## Version 1 10/28/2021

In-Press Preview

## Version 2 12/08/2021

Electronic publication
