# Supplementary material for: A potential pathogenic association between periodontal disease and Crohn’s disease
Source: JCI Insight. 2021 Dec 8;6(23):e148543. doi: 10.1172/jci.insight.148543 (PMC8675195; doi:10.1172/jci.insight.148543)
Supplement: Supplemental data [file jciinsight-6-148543-s179.pdf]

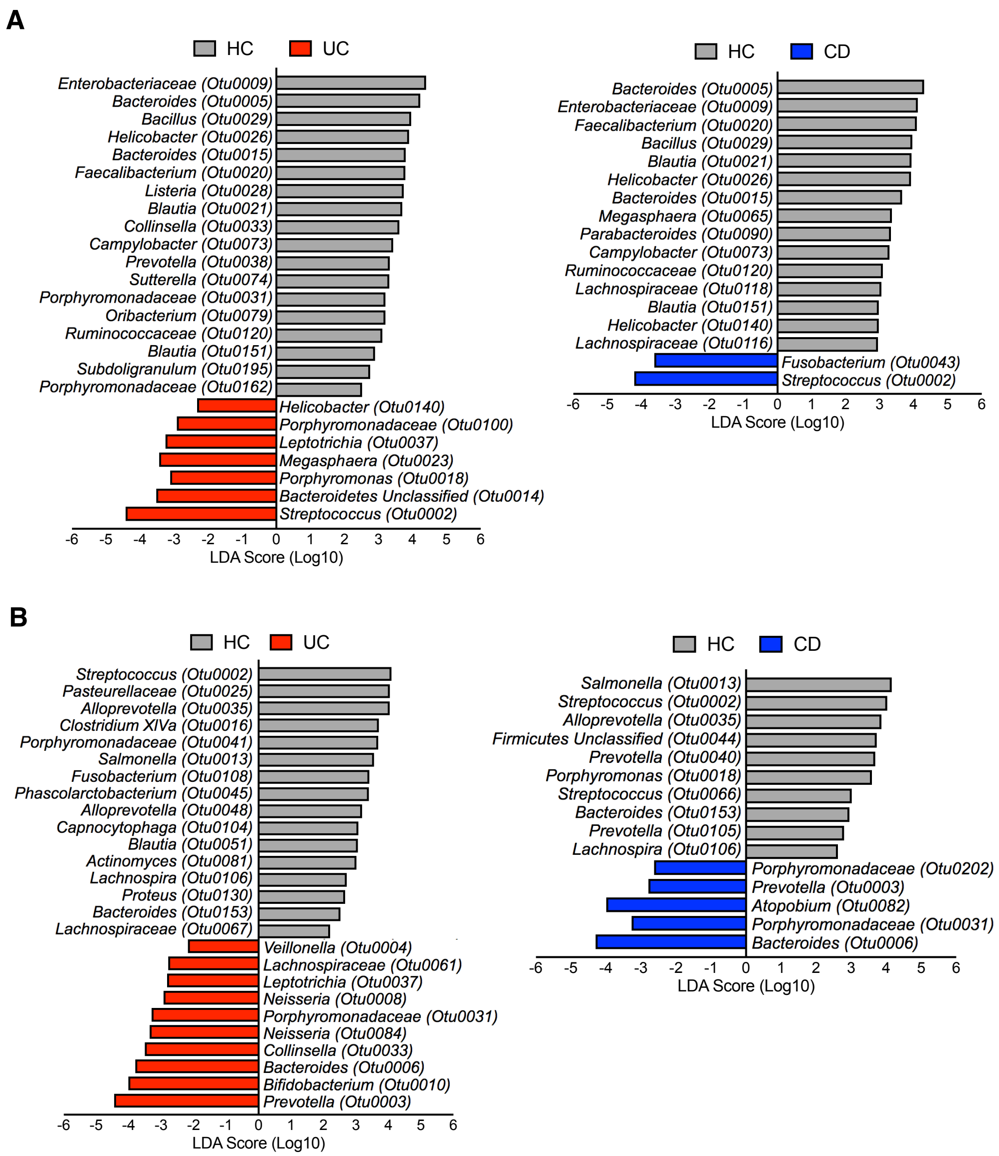

Supplemental Figure 1. Bacterial taxa differentially abundant in IBD.
Bacterial taxa significantly enriched in UC or CD compared to HC were identified by LEfSe. (A) Oral microbiota (saliva). (B) Gut microbiota (stool).

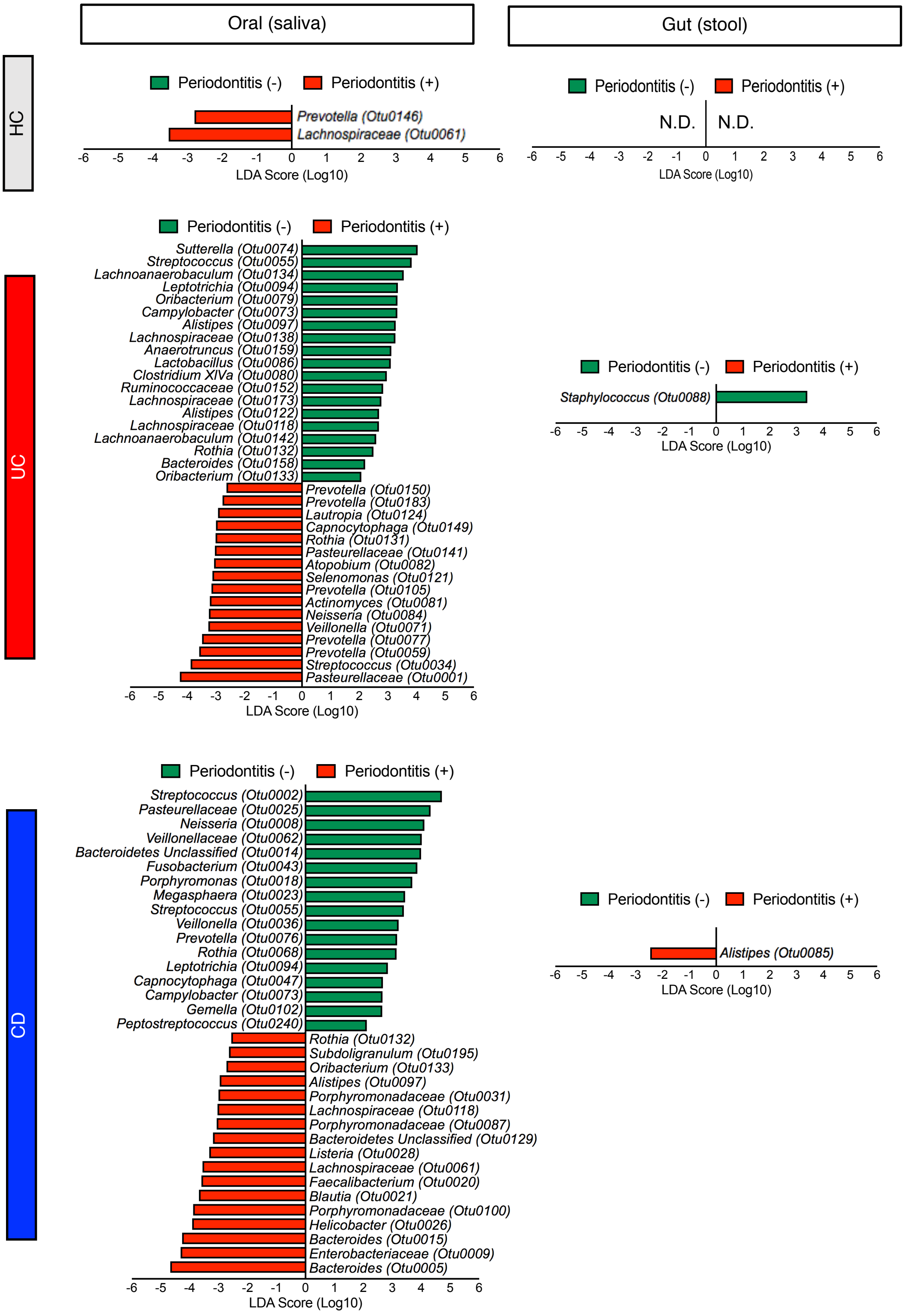

Supplemental Figure 2. Bacterial taxa differentially abundant in incipient periodontitis. Bacterial taxa significantly different between the presence and absence of incipient periodontitis were identified by LEfSe. (A) Oral microbiota (saliva). (B) Gut microbiota (stool). N.D.; not detected

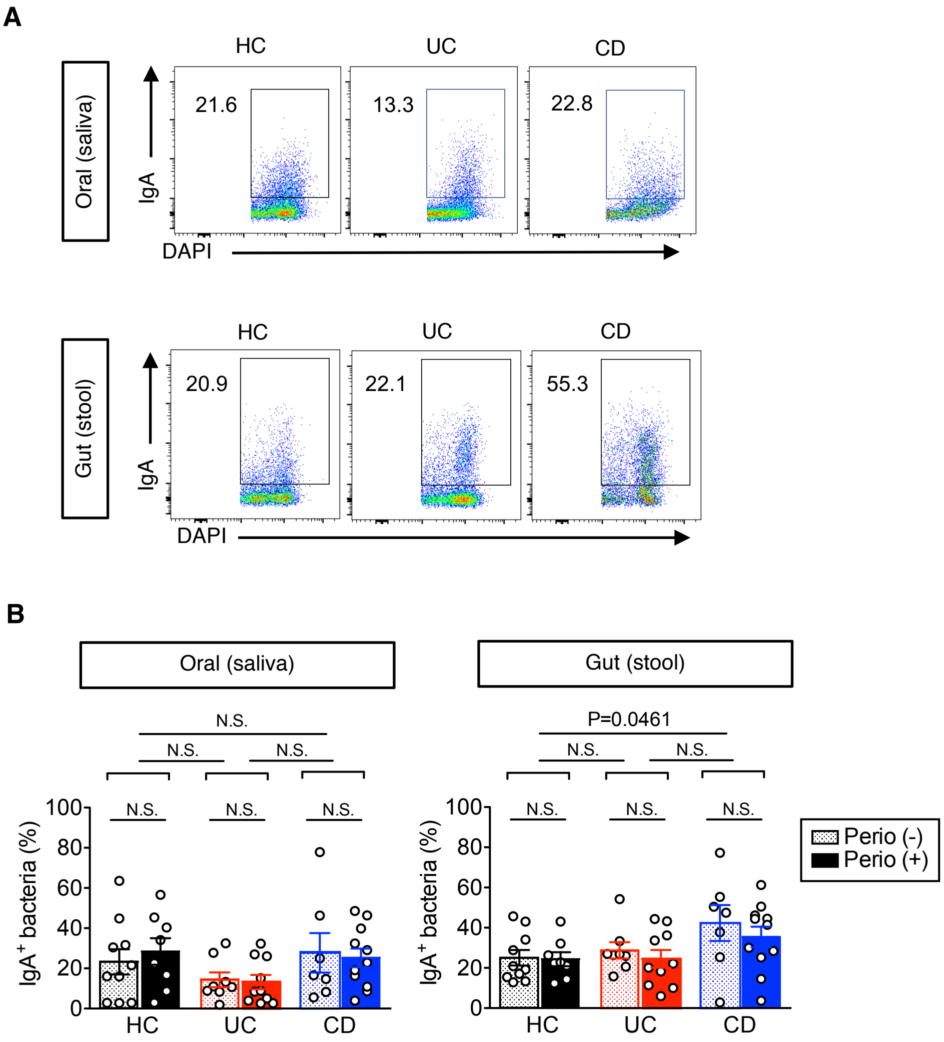

Supplemental Figure 3. IgA-coated bacteria in the saliva and stool.
The abundance of IgA-coated bacteria in the stool. (A) Representative FACS plots. (B) Statistical analysis. Results are shown as mean ± SD. Dots indicate individual participants (HC = 18, UC = 18, CD = 18). Statistically significant *P* value by 1-way ANOVA followed by Bonferroni post hoc test is shown. N.S., not significant.

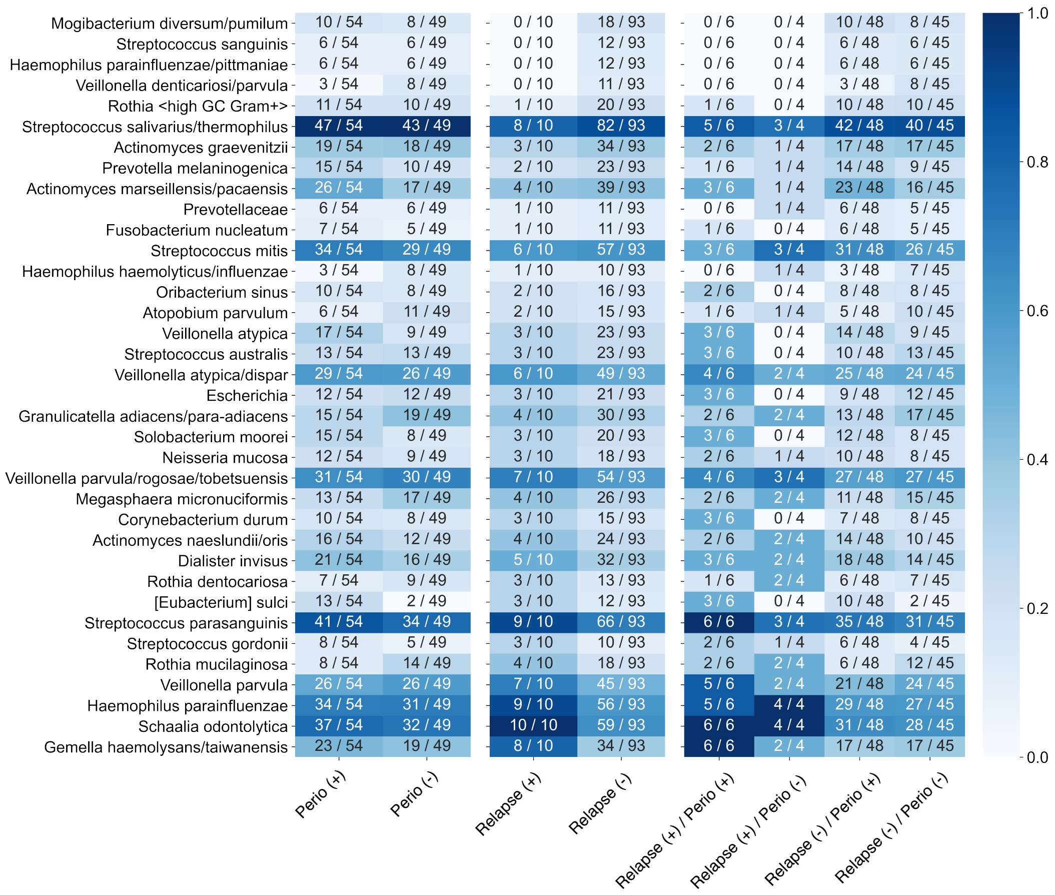

Supplemental Figure 4. Prevalence of bacterial species shared between the oral and gut microbiome.

The prevalence of bacterial species commonly colonized in the oral and gut mucosa is shown.

Supplemental Table1. Oral characteristics of study participants

Group		ID#	Age	Smoking	Number of teeth	Decayed teeth	Missing teeth	Filled teeth	Retinal teeth	Wisdom teeth	Oral moisture degree	Bleeding on Probing: Maxillary Right 4-7	Bleeding on Probing: Maxillary 3-3	Bleeding on Probing: Maxillary Left 4-7	Bleeding on Probing: Mandibular Right 4-7	Bleeding on Probing: Mandibular 3-3	Bleeding on Probing: Mandibular Left 4-7	Periodontal pocket >4mm: Maxillary Right 4-7	Periodontal pocket >4mm: Maxillary 3-3	Periodontal pocket >4mm: Maxillary Left 4-7	Periodontal pocket >4mm: Mandibular Right 4-7	Periodontal pocket >4mm: Mandibular 3-3	Periodontal pocket >4mm: Mandibular Left 4-7	Note
HC	Periodontitis (+)	HC-03	29	Current	28	0	0	4	0	0	30.7	4	6	4	4	6	4	1	0	1	1	0	1	
		HC-05	36	None	28	0	0	3	0	0	31.0	2	0	0	0	0	0	0	0	0	0	0	1	
		HC-09	24	None	28	4	0	8	4	4	30.0	3	6	4	4	3	3	1	1	1	3	0	2	
		HC-10	22	Current	28	0	0	5	2	2	29.8	2	3	3	2	3	3	0	0	1	1	0	1	
		HC-11	36	None	28	0	0	13	0	0	31.0	2	0	0	1	1	1	1	0	1	1	0	1	
		HC-12	35	None	28	0	0	10	0	0	30.3	4	5	4	2	5	4	0	0	0	2	0	0	
		HC-17	28	None	28	0	0	5	3	4	30.9	1	2	2	0	2	2	0	0	0	1	0	0	
		HC-18	23	None	28	0	0	1	2	4	31.0	1	0	3	0	4	0	2	0	1	0	0	0	
		HC-19	21	None	28	0	0	0	2	4	31.5	3	0	0	0	6	0	1	0	2	1	0	1	
		HC-23	23	None	28	1	0	6	0	1	31.9	0	5	0	3	5	0	0	0	0	1	0	0	
		HC-25	30	None	28	0	0	11	0	1	29.0	4	2	1	3	0	0	1	2	1	1	0	0	
		HC-26	31	None	28	2	0	11	0	4	30.9	4	2	1	3	0	0	1	2	1	1	0	0	
		HC-27	37	Current	28	0	0	15	4	4	26.4	0	0	1	1	0	2	0	0	0	1	0	0	
		HC-28	36	Current	28	3	0	8	0	3	29.6	1	1	0	2	0	0	0	0	0	2	1	2	
		HC-31	35	None	28	0	0	4	2	3	27.0	3	2	3	1	0	1	0	0	1	0	0	0	
		HC-33	31	None	28	0	0	5	0	0	29.3	1	1	2	3	1	2	1	0	1	0	0	0	
		HC-36	29	None	27	7	1*	5	0	2	31.8	5	6	4	2	2	2	0	0	0	1	0	1	*; Tooth extraction due to malocclusion
		HC-37	25	None	28	1	0	6	0	1	33.6	2	6	4	4	4	3	2	0	0	1	0	2	
		HC-39	38	None	28	0	0	5	1	3	30.7	2	0	3	2	1	1	0	0	0	1	0	1	
		HC-42	29	None	28	1	0	6	2	4	31.2	4	4	4	3	1	3	0	0	0	1	0	1	
		HC-43	32	None	28	0	0	6	0	0	31.5	4	4	3	3	2	2	0	0	0	1	0	0	
		HC-48	36	Current	28	3	0	11	0	0	27.1	1	0	1	2	2	0	0	0	0	1	0	0	
	Periodontitis (-)	HC-01	39	Ex	28	0	0	17	3	4	30.6	0	0	0	0	0	0	0	0	0	0	0	0	
		HC-02	42	None	28	0	0	6	0	0	29.8	2	1	3	1	1	1	0	0	0	0	0	0	
		HC-04	24	None	28	0	0	0	4	4	28.3	0	0	0	0	0	1	0	0	0	0	0	0	
		HC-07	37	None	28	0	0	2	0	0	25.6	0	0	2	1	1	0	0	0	0	0	0	0	
		HC-08	27	None	28	0	0	10	0	0	22.5	3	6	3	4	4	4	0	0	0	0	0	0	
		HC-14	32	None	28	0	0	4	0	0	30.1	0	0	0	0	0	0	0	0	0	0	0	0	
		HC-16	30	None	28	0	0	10	0	0	31.3	1	1	3	2	1	0	0	0	0	0	0	0	
		HC-20	23	None	28	0	0	0	3	3	29.4	0	0	4	4	3	4	0	0	0	0	0	0	
		HC-21	33	None	28	0	0	14	0	0	28.1	1	0	2	1	0	0	0	0	0	0	0	0	
		HC-22	24	None	28	0	0	0	0	0	30.2	3	0	2	0	2	1	0	0	0	0	0	0	
		HC-24	36	None	28	0	0	4	0	0	33.4	2	2	4	3	2	3	0	0	0	0	0	0	
		HC-29	34	Ex	28	1	0	8	0	0	27.6	3	1	2	1	2	2	0	0	0	0	0	0	
		HC-30	36	None	28	0	0	10	0	0	32.4	0	0	0	0	0	0	0	0	0	0	0	0	
		HC-32	36	None	28	0	0	13	0	0	31.4	3	2	2	1	3	1	0	0	0	0	0	0	
		HC-34	32	Ex	28	0	0	5	2	2	33.0	0	0	0	0	0	0	0	0	0	0	0	0	
		HC-35	28	None	28	1	0	0	0	4	22.5	0	0	3	2	1	1	0	0	0	0	0	0	
		HC-40	28	None	27	0	1*	7	1	1	30.5	1	0	2	1	1	2	0	0	0	0	0	0	*; Tooth extraction due to malocclusion
		HC-44	35	None	28	0	0	0	0	2	29.5	2	6	3	3	2	4	0	0	0	0	0	0	
		HC-45	25	None	27	0	1*	10	0	0	30.7	1	4	3	2	4	0	0	0	0	0	0	0	*; Tooth extraction due to malocclusion
		HC-46	27	Current	28	0	0	6	4	4	30.1	0	0	0	0	0	0	0	0	0	0	0	0	
		HC-47	35	None	28	0	0	15	0	0	50.9	2	3	4	1	2	1	0	0	0	0	0	0	
		HC-49	35	None	28	7	0	8	0	2	33.1	1	4	2	0	0	1	0	0	0	0	0	0	
		HC-50	31	None	28	0	0	7	0	0	23.3	1	1	2	1	0	2	0	0	0	0	0	0	

Group			Age	Smoking	Number of teeth	Decayed teeth	Missing teeth	Filled teeth	Retinal teeth	Wisdom teeth	Oral moisture degree	Bleeding on Probing: Maxillary Right 4-7	Bleeding on Probing: Maxillary 3-3	Bleeding on Probing: Maxillary Left 4-7	Bleeding on Probing: Mandibular Right 4-7	Bleeding on Probing: Mandibular 3-3	Bleeding on Probing: Mandibular Left 4-7	Periodontal pocket >4mm: Maxillary Right 4-7	Periodontal pocket >4mm: Maxillary 3-3	Periodontal pocket >4mm: Maxillary Left 4-7	Periodontal pocket >4mm: Mandibular Right 4-7	Periodontal pocket >4mm: Mandibular 3-3	Periodontal pocket >4mm: Mandibular Left 4-7	Note
UC	Periodontitis (+)	UC-01	23	Current	28	0	0	0	1	2	30.6	3	1	3	3	2	0	1	0	0	0	0	0	
		UC-11	29	None	28	3	0	8	0	4	33.1	0	0	0	0	0	0	0	0	0	1	0	0	
		UC-12	19	None	28	0	0	0	4	4	30.8	4	4	4	3	6	1	0	0	1	1	0	0	
		UC-14	34	None	28	0	0	0	2	3	29.2	3	0	3	0	1	2	0	0	1	0	0	1	
		UC-15	38	None	28	0	0	13	2	3	30.8	4	2	3	2	0	2	1	0	0	1	0	1	
		UC-16	17	None	28	0	0	0	4	4	31.9	0	0	0	0	0	0	0	0	1	0	0	0	
		UC-20	40	None	28	0	0	20	0	2	30.3	4	4	4	1	0	1	3	0	2	1	0	2	
		UC-21	37	None	28	0	0	7	1	4	31.7	0	0	0	0	0	0	0	0	1	0	0	0	
		UC-23	23	None	28	0	0	0	3	4	19.8	0	0	3	1	0	0	0	0	1	0	0	0	
		UC-24	35	None	28	0	0	8	2	2	30.0	2	0	1	0	0	0	2	0	1	0	0	0	
		UC-26	26	None	28	0	0	5	0	0	31.4	1	6	2	2	0	0	0	0	1	0	0	0	
		UC-27	19	None	28	0	0	4	4	4	31.9	3	2	3	3	3	0	0	0	0	1	0	0	
		UC-29	32	Current	28	0	0	5	0	0	25.2	0	0	0	1	0	1	0	0	0	1	0	1	
		UC-31	40	None	28	1	0	2	0	4	26.6	0	0	1	0	0	1	0	0	0	1	0	2	
		UC-34	39	None	28	0	0	5	0	0	31.4	0	6	3	1	0	1	0	0	0	1	0	1	
		UC-35	40	None	28	2	0	1	0	2	30.1	4	3	1	2	2	3	0	0	0	1	0	0	
		UC-36	32	None	28	10	0	3	2	4	29.8	0	0	1	4	0	0	0	0	1	1	0	0	
		UC-39	27	None	28	0	0	10	0	0	28.2	0	0	3	1	0	0	0	0	0	1	0	0	
		UC-41	26	None	28	0	0	9	0	3	28.5	1	0	3	5	4	2	0	0	0	1	0	0	
		UC-42	25	None	28	1	0	2	0	0	30.4	0	0	0	2	1	0	0	0	0	0	0	1	
		UC-43	35	None	27	2	1*	9	0	3	32.9	4	2	4	1	6	1	1	0	0	0	0	0	*; Tooth extraction due to malocclusion
		UC-44	20	None	28	1	0	1	1	4	27.2	0	0	2	0	0	0	0	0	0	0	0	1	
		UC-45	26	None	28	0	0	6	2	4	27.9	4	6	4	4	6	4	0	0	1	2	0	1	
	Periodontitis (-)	UC-02	35	None	28	1	0	10	2	3	32.8	0	0	0	0	1	0	0	0	0	0	0	0	
		UC-03	30	None	28	0	0	4	4	4	31.0	0	1	0	1	1	1	0	0	0	0	0	0	
		UC-04	22	None	28	0	0	0	0	1	32.2	2	2	1	2	0	0	0	0	0	0	0	0	
		UC-05	25	None	28	0	0	1	3	4	29.9	0	0	0	0	0	0	0	0	0	0	0	0	
		UC-06	34	None	28	0	0	3	0	0	28.8	0	0	3	0	2	0	0	0	0	0	0	0	
		UC-08	32	None	28	0	0	10	0	0	30.5	0	0	0	0	0	0	0	0	0	0	0	0	
		UC-09	39	None	28	0	0	5	0	0	25.5	0	0	0	0	0	0	0	0	0	0	0	0	
		UC-10	26	None	28	3	0	3	0	1	30.7	1	2	2	0	2	2	0	0	0	0	0	0	
		UC-13	28	Current	28	0	0	7	0	4	33.8	2	1	3	2	6	1	0	0	0	0	0	0	
		UC-17	22	Current	28	0	0	1	4	4	31.0	4	3	4	4	4	4	0	0	0	0	0	0	
		UC-18	22	None	28	1	0	0	4	4	21.2	0	0	2	0	0	0	0	0	0	0	0	0	
		UC-19	17	None	28	0	0	1	0	0	31.9	1	0	1	0	0	1	0	0	0	0	0	0	
		UC-22	36	Current	28	1	0	14	1	1	32.0	1	4	3	4	4	1	0	0	0	0	0	0	
		UC-30	29	None	28	0	0	0	0	4	33.5	2	1	4	1	0	2	0	0	0	0	0	0	
		UC-32	38	None	27	0	1*	10	0	0	31.7	0	0	0	0	0	0	0	0	0	0	0	0	*; Tooth extraction due to malocclusion
		UC-33	23	None	28	1	0	9	2	4	31.8	0	0	0	0	0	0	0	0	0	0	0	0	
		UC-37	30	None	28	0	0	10	1	1	30.5	0	1	2	0	0	0	0	0	0	0	0	0	
		UC-38	38	None	28	0	0	0	1	2	31.6	1	0	2	0	0	1	0	0	0	0	0	0	
		UC-40	20	None	28	0	0	0	4	4	28.8	2	0	2	1	2	1	0	0	0	0	0	0	

Group			Age	Smoking	Number of teeth	Decayed teeth	Missing teeth	Filled teeth	Retinal teeth	Wisdom teeth	Oral moisture degree	Bleeding on Probing: Maxillary Right 4-7	Bleeding on Probing: Maxillary 3-3	Bleeding on Probing: Maxillary Left 4-7	Bleeding on Probing: Mandibular Right 4-7	Bleeding on Probing: Mandibular 3-3	Bleeding on Probing: Mandibular Left 4-7	Periodontal pocket >4mm: Maxillary Right 4-7	Periodontal pocket >4mm: Maxillary 3-3	Periodontal pocket >4mm: Maxillary Left 4-7	Periodontal pocket >4mm: Mandibular Right 4-7	Periodontal pocket >4mm: Mandibular 3-3	Periodontal pocket >4mm: Mandibular Left 4-7	Note
CD	Periodontitis (+)	CD-01	33	None	27	2	1*	13	1	4	28.4	2	1	3	3	1	1	1	0	0	0	0	0	*; Tooth extraction due to malocclusion
		CD-02	18	None	28	0	0	0	4	4	30.8	1	2	0	0	0	0	0	0	0	1	0	0	
		CD-03	40	None	28	4	0	0	0	2	29.3	4	2	3	3	1	2	1	0	1	1	0	0	
		CD-07	26	None	28	0	0	15	0	0	30.7	0	6	4	4	3	4	0	0	0	1	0	0	
		CD-10	24	None	28	15	0	3	0	4	32.3	2	6	4	4	6	5	0	0	0	0	0	1	
		CD-12	18	None	28	4	0	7	4	4	28.9	0	2	3	2	0	3	0	0	0	1	0	1	
		CD-13	33	None	28	0	0	12	0	0	30.0	2	4	4	4	4	4	0	0	1	0	0	0	
		CD-16	20	None	28	0	0	12	0	0	27.5	2	2	2	2	1	2	0	0	0	0	1	0	
		CD-17	24	None	28	1	0	2	3	4	27.5	0	0	0	2	2	2	0	0	0	0	0	1	
		CD-19	26	None	28	0	0	3	0	4	21.6	2	2	3	2	2	4	1	1	2	1	0	2	
		CD-22	34	None	28	2	0	1	4	4	34.8	0	0	2	2	4	1	0	0	1	0	0	0	
	Periodontitis (-)	CD-06	22	Current	28	0	0	0	2	2	31.0	1	0	2	1	4	1	0	0	0	0	0	0	
		CD-08	25	None	28	0	0	1	0	2	31.0	1	0	2	1	4	1	0	0	0	0	0	0	
		CD-14	27	None	28	0	0	0	2	3	34.2	3	6	5	4	6	4	0	0	0	0	0	0	
		CD-18	26	None	28	1	0	14	0	0	30.4	1	0	1	0	0	0	0	0	0	0	0	0	
		CD-20	32	None	28	0	0	1	0	1	20.3	0	0	0	0	0	0	0	0	0	0	0	0	
		CD-24	34	Ex	28	0	0	10	1	1	26.1	0	0	0	0	0	0	0	0	0	0	0	0	
		CD-25	19	None	28	0	0	0	0	2	27.9	2	1	3	0	0	0	0	0	0	0	0	0	

Supplymental Table 2. Clinical features of patients with inflammatory bowel disease

		No.	Mayo score	Disease duration (years)	Disease extent	Steroid	Relapse	Extraintestinal complications (EIM)							Treatment history				First diagnosis
								PBC	PSC	Gallstone	Spondyloarthritis	Arthritis	Aphthous	Erythema nodosum	5-ASA	thioprine	αTNF	PSL	
UC	Periodontitis (+)	UC-01	4	7.6	E3	SR									○	○	○		2010-12
		UC-11	1	4.3	E1	SF									○				2014-03
		UC-12	1	5.8	E3	SD	○								○	○		○	2012-08
		UC-14	2	6.2	E3	SF									○				2012-05
		UC-15	0	4.7	E3	SF									○				2013-11
		UC-16	5	2	E3	SF						○			○				2016-07
		UC-20	1	3.3	E3	SF									○				2015-04
		UC-21	1	8.9	E3	SF									○				2009-10
		UC-23	3	6.6	E3	SD									○	○		○	2011-11
		UC-24	3	0.4	E3	SD									○			○	2018-03
		UC-26	0	12.7	E3	SF								○					2005-11
		UC-27	1	2.1	E2	SF	○							○					2016-06
		UC-29	0	4.2	E3	SF						○			○				2014-06
		UC-31	1	2.1	E3	SF									○				2016-09
		UC-34	1	8.6	E1	SF									○				2010-01
		UC-35	2	16.6	E2	SF									○	○			2002-02
		UC-36	2	11.5	E3	SF			○						○				2007-02
		UC-39	5	9.7	E2	SD	○								○	○		○	2009-07
		UC-41	2	7.4	E2	SF									○				2011-05
		UC-42	4	5	E3	SF									○	○	○		2013-09
		UC-43	1	15.1	E3	SF									○				2003-08
		UC-44	0	0.3	E3	SF													2018-05
		UC-45	0	9.2	E1	SF									○				2009-11
	Periodontitis (-)	UC-02	3	19.9	E3	SD						○			○	○		○	1998-07
		UC-03	2	1.4	E3	SD	○					○		○	○	○			2017-02
		UC-04	3	4	E3	SD								○	○		○	○	2014-06
		UC-05	2	8.8	E3	SF									○				2009-10
		UC-06	4	4.7	E3	SF									○				2013-11
		UC-08	0	2.6	E2	SF									○				2015-11
		UC-09	1	1.1	E3	SF	○								○				2017-04
		UC-10	2	1.6	E1	SF									○			○	2016-11
		UC-13	0	11.4	E3	SF									○				2007-02
		UC-17	0	4.1	E3	SD									○	○	○		2014-05
		UC-18	2	8.3	E3	SD									○	○			2010-03
		UC-19	5	0.5	E3	SD						○			○	○		○	2017-12
		UC-22	1	2.4	E3	SF									○				2016-03
		UC-30	1	1.7	E2	SF									○				2016-11
		UC-32	3	9.2	E2	SF									○				2009-06
		UC-33	0	4.4	E3	SF	○								○			○	2014-04
		UC-37	0	5.4	E3	SF			○						○				2013-03
		UC-38	2	13.9	E2	SF	○								○				2004-11
		UC-40	0	6.3	E3	SF			○						○	○			2012-06

Extent: E1; Ulcerative proctitis, E2; Left-side UC, E3; Extensive
Steroid: Steroid refractory (SR), Steroid dependent (SD), Steroid free (SF)

	CD	No.	Simple CDAI	Disease duration (years)	Disease location	Disease behavior	Resistance to Biologics (RE)	Relapse	Extraintestinal complications (EIM)							Treatment history				First diagnosis
									PSC	Pancreatitis	Gallstone	Spondyloarthritis	Arthritis	Aphthous	Erythema nodosum	5-ASA	thioprine	αTNF	PSL	
CD	Periodontitis (+)	CD-01	1	15.1	L1	B1										○	○	○		2003-06
		CD-02	1	2.3	L3	B2		○								○		○		2016-04
		CD-03	0	1.9	L3	B2		○								○			○	2016-08
		CD-07	0	2.1	L1	B1							○			○	○			2016-07
		CD-10	0	6	L3	B2											○	○		2012-08
		CD-12	0	1.9	L3	B2										○	○			2016-08
		CD-13	3	17.1	L3	B2										○	○	○		2001-06
		CD-16	2	3.5	L1	B2										○		○		2015-02
		CD-17	0	3.2	L3	B1										○		○		2015-06
		CD-19	0	4.6	L3	B2				○						○	○	○		2014-01
		CD-22	1	1.1	L3	B2		○								○	○		○	2017-07
	Periodontitis (-)	CD-06	7	6.7	L3	B3								○					○	2011-09
		CD-08	0	7.3	L2	B1										○				2011-05
		CD-14	0	6.4	L3	B2										○	○	○		2012-02
		CD-18	0	14.6	L3	B1										○		○		2004-02
		CD-20	6	8.8	L1	B2										○		○		2009-11
		CD-24	1	17.5	L3	B1								○		○				2001-05
		CD-25	0	4	L3	B2										○				2015-01

Location (L): L1; Terminal ileum, L2; Colon, L3: Ileocolon, L4; Upper GI
Behaviour (B): B1; Non-stricturing, non-penetrating, B2; Stricturing, B3; Penetrating

Supplymental Table 3. Clinical course of patients with inflammatory bowel disease

			Initial (day 0)		Follo-up visit (1st)					Follo-up visit (2nd)					Follo-up visit (3rd)					
			Dental check (mm/dd/yy)	pMayo	Visit date (mm/dd/yy)	Interval (days from initial)	pMayo	Add drug	Relapse	Visit date (mm/dd/yy)	Interval (days from initial)	pMayo	Add drug	Relapse	Visit date (mm/dd/yy)	Interval (days from initial)	pMayo	Add drug	Relapse	Composite
UC	Periodontitis (+)	UC-01	07/09/18	4	09/29/18	82	2			12/04/18	148	1			02/03/19	209	1			+
		UC-11	07/09/18	1	09/03/18	56	1			10/15/18	98	0			11/12/18	126	0			+
		UC-12	06/19/18	1	07/03/18	14	0			08/14/18	56	0			10/09/18	112	4	PSL+	+	+
		UC-14	07/24/18	2	09/18/18	56	2			11/20/18	119	2			01/08/19	168	2			
		UC-15	07/31/18	0	10/02/18	63	3			12/18/18	140	0			02/25/19	209	0			+
		UC-16	07/30/18	5	09/24/18	56	3			11/04/18	97	2			01/12/19	166	1			
		UC-20	08/02/18	1	09/20/18	49	0			11/15/18	105	0			01/17/19	168	0			+
		UC-21	08/27/18	1	10/29/18	63	0			12/20/18	115	0			02/21/19	178	0			+
		UC-23	06/30/18	3	09/04/18	66	1			12/03/18	156	1			02/05/19	220	1			+
		UC-24	08/25/18	3	11/04/18	71	1			01/12/19	140	1			03/22/19	209	1			+
		UC-26	07/17/18	0	08/14/18	28	0			09/11/18	56	6			09/18/18	63	2			+
		UC-27	07/17/18	1	09/10/18	55	3	5-ASA up	+	11/02/18	108	2			02/03/19	201	1		+	+
		UC-29	08/21/18	0	10/16/18	56	0			12/11/18	112	0			02/05/19	168	0			+
		UC-31	10/11/18	1	12/06/18	56	1			02/03/19	115	1			04/26/19	197	0			+
		UC-34	09/04/18	1	11/13/18	70	0			01/29/19	147	0			03/12/19	189	0			+
		UC-35	09/04/18	2	11/06/18	63	2			01/22/19	140	2			03/26/19	203	2			
		UC-36	09/08/18	2	11/15/18	68	2			01/26/19	140	2			04/13/19	217	2			
		UC-39	03/20/19	5	05/14/19	55	0			07/09/19	111	0			07/22/19	124	4	rectum 5-ASA	+	+
		UC-41	09/22/18	2	11/24/18	63	2			01/26/19	126	2			03/30/19	189	2			
		UC-42	10/02/18	4	11/27/18	56	0			01/22/19	112	0			03/19/19	168	0			
		UC-43	09/06/18	1	10/18/18	42	0			11/15/18	70	0			01/10/19	126	3			+
		UC-44	09/18/18	0	12/22/18	95	0			02/04/19	139	0			04/27/19	221	0			+
		UC-45	01/21/19	0	03/18/19	56	0			05/27/19	126	0			07/22/19	182	0			+
	Periodontitis (-)	UC-02	05/31/18	3	07/22/18	52	2			09/24/18	116	0			12/13/18	196	0			
		UC-03	07/10/18	2	09/04/18	56	3	SASP+	+	11/26/18	139	1			02/03/19	208	0			+
		UC-04	06/12/18	3	09/03/18	83	2			11/20/18	161	1			02/03/19	236	0			
		UC-05	07/28/18	2	10/03/18	67	1			12/11/18	136	0			02/06/19	193	0			+
		UC-06	07/28/18	4	09/29/18	63	2			10/27/18	91	2			11/27/18	122	4			
		UC-08	07/13/18	0	08/06/18	24	0			09/04/18	53	0			10/29/18	108	1			+
		UC-09	06/04/18	1	07/03/18	29	4	5-ASA up	+	08/25/18	82	2			10/04/18	122	1			+
		UC-10	06/26/18	2	09/03/18	69	0			12/04/18	161	0			02/06/19	225	0			
		UC-13	07/30/18	0	10/01/18	63	0			12/03/18	126	0			01/21/19	175	0			+
		UC-17	07/02/18	0	08/20/18	49	0			10/01/18	91	0			12/17/18	168	0			+
		UC-18	06/30/18	2	08/21/18	52	0			10/16/18	108	0			11/20/18	143	1			
		UC-19	06/11/18	5	07/08/18	27	3			10/03/18	114	2			12/26/18	198	1			
		UC-22	08/30/18	1	10/25/18	56	0			11/29/18	91	0			02/14/19	168	1			+
		UC-30	07/17/18	1	09/11/18	56	0			10/30/18	105	0			12/17/18	153	0			+
		UC-32	08/28/18	3																
		UC-33	09/10/18	0	11/19/18	70	0			01/21/19	133	5	rectum 5-ASA	+	02/25/19	168	3		+	+
		UC-37	08/14/18	0	09/22/18	39	2			11/22/18	100	1			01/15/19	154	1			+
		UC-38	09/25/18	2	11/27/18	63	6	PSL up	+											+
		UC-40	09/19/18	0	12/05/18	77	0			02/19/19	153	0								+

			Initial (day 0)		Follo-up visit (1st)					Follo-up visit (2nd)					Follo-up visit (3rd)					
			Dental check (mm/dd/yy)	pMayo	Visit date (mm/dd/yy)	Interval (days from initial)	pMayo	Add drug	Relapse	Visit date (mm/dd/yy)	Interval (days from initial)	pMayo	Add drug	Relapse	Visit date (mm/dd/yy)	Interval (days from initial)	pMayo	Add drug	Relapse	Composite
CD	Periodontitis (+)	CD-01	07/04/18	1	08/29/18	56	0			10/24/18	112	0			12/19/18	168	0			
		CD-02	07/30/18	1	08/13/18	14	6	aTNF up	+	09/15/18	47	5			11/04/18	97	4			+
		CD-03	07/10/18	0	09/04/18	56	0			11/06/18	119	5	PSL +	+	12/16/18	159	3			+
		CD-07	08/13/18	0	10/09/18	57	0			12/04/18	113	0			02/05/19	176	0			
		CD-10	08/14/18	0	10/09/18	56	0			12/18/18	126	0			01/18/19	157	0			
		CD-12	07/31/18	0	09/18/18	49	0			10/23/18	84	0			01/13/19	166	1			+
		CD-13	08/08/18	3	09/25/18	48	0			11/14/18	98	0			01/09/19	154	1			+
		CD-16	08/28/18	2	10/23/18	56	2			12/18/18	112	0			02/12/19	168	2			+
		CD-17	08/21/18	0	10/16/18	56	0			12/04/18	105	0			01/29/19	161	0			
		CD-19	08/29/18	0	10/31/18	63	0			12/26/18	119	0			02/08/19	163	0			
		CD-22	08/25/18	1	09/29/18	35	3			11/01/18	68	5	PSL +	+	02/18/19	177	2			+
	Periodontitis (-)	CD-06	06/12/18	7	09/12/18	92	4			11/15/18	156	3			01/25/19	227	1			
		CD-08	09/08/18	0	12/08/18	91	0			03/09/19	182	0			05/30/19	264	0			
		CD-14	07/28/18	0	10/13/18	77	0			12/08/18	133	0			02/03/19	190	0			
		CD-18	09/22/18	0	10/27/18	35	0			12/22/18	91	0			03/09/19	168	0			
		CD-20	08/22/18	6	11/02/18	72	0			02/14/19	176	0			05/25/19	276	1			+
		CD-24	11/05/18	1	01/28/19	84	0			04/22/19	168	0			07/23/19	260	0			
		CD-25	01/08/19	0	03/05/19	56	2			06/18/19	161	0			08/13/19	217	0			+
